# Upregulation of key genes Eln and Tgfb3 were associated with the severity of cardiac hypertrophy

**DOI:** 10.1186/s12864-022-08778-0

**Published:** 2022-08-14

**Authors:** Rui Zhang, Xuan Xu, Xi Chen, Chunshu Hao, Zhenjun Ji, Pengfei Zuo, Mingming Yang, Genshan Ma, Yongjun Li

**Affiliations:** Department of Cardiology, Zhongda Hospital, School of Medicine, Southeast University, 210009 Nanjing, P. R. China

**Keywords:** Cardiac hypertrophy, Bioinformatics, GEO database, Key gene

## Abstract

**Background:**

Hypertension-induced cardiac hypertrophy is one of the most common pre-conditions that accompanies heart failure. This study aimed to identify the key pathogenic genes in the disease process.

**Methods:**

GSE18224 was re-analyzed and differentially expressed genes (DEGs) were obtained. Gene Ontology (GO) and Kyoto Encyclopedia of Genes and Genomes (KEGG) analyses were carried out. Networks of transcription factor (TF)-mRNA, microRNA (miRNA)-mRNA and Protein-Protein interaction (PPI) were constructed, and a key module was further screened out from PPI network. GSE36074 dataset and our transverse aortic constriction (TAC) mouse model were used to validate gene expression in the module. Finally, the correlation between the genes and biomarkers of cardiac hypertrophy were evaluated.

**Results:**

Totally, there were 348 DEGs in GSE18224, which were mainly enriched in biological processes including collagen fibril organization, cellular response to transforming growth factor-beta stimulus and were involved in ECM-receptor interaction and Oxytocin signaling pathway. There were 387 miRNAs targeted by 257 DEGs, while 177 TFs targeted 71 DEGs. The PPI network contained 222 nodes and 770 edges, with 18 genes screened out into the module. After validation, 8 genes, which were also significantly upregulated in the GSE36074 dataset, were selected from the 18 DEGs. 2 of the 8 DEGs, including Eln and Tgfb3 were significantly upregulated in our mouse model of myocardial hypertrophy. Finally, the expression of Eln and Tgfb3 were found to be positively correlated with the level of the disease biomarkers.

**Conclusions:**

Upregulated key genes Eln and Tgfb3 were positively correlated with the severity of cardiac hypertrophy, which may provide potential therapeutic targets for the disease.

**Supplementary Information:**

The online version contains supplementary material available at 10.1186/s12864-022-08778-0.

## Introduction

Cardiovascular diseases (CVDs) have occupied a major position in disability and death all around the world. In general, cardiac hypertrophy is especially common among all the CVDs, and the main structural feature is the significant increase in cardiomyocyte size and heart mass [1]. Cardiac hypertrophy is usually regarded as an adaptive response of the heart to multiple physiological or pathological stimulations. Its internal characteristic features contain the following: enhanced protein synthesis and expression of fetal genes such as natriuretic peptide A (NPPA), natriuretic peptide B (NPPB) and myosin heavy chain 7 (MYH7), which encode atrial natriuretic peptide (ANP), brain natriuretic peptide (BNP) and myosin heavy chain, cardiac muscle β-isoform (MYHCβ) respectively [2]. Initially, the hypertrophic heart can still maintain normal output despite facing pressure overload. Once myocardial hypertrophy becomes established, it will bring serious adverse prognoses such as functional failure, arrhythmia, and even sudden death [3].

The hypertrophic process usually results from biomechanical and stretch-sensitive stimuli, or neurohormonal stimuli [4]. Over the past decades, several signaling pathways have been discovered to mediate the development of cardiac hypertrophy, including Ca2+/calmodulin, mitogen-activated protein kinase (MAPK), janus kinase/signal transducer and activator of tran-ions (JAK‐STAT), phosphatidylinositol 3‐kinase (PI3K)/Akt, nuclear factor‐κB (NF‐κB) and adenosine‐activated protein kinase (AMPK) [5]. These signaling pathways are activated by various external pro-hypertrophic stimulations and the subsequent crosstalk between the signaling pathways mediates the pathological response and further promotes signal transduction into the nucleus to transcriptionally regulate gene expression, eventually leading to the progression of hypertrophy [6]. Based on the above research results, strategies have been developed to treat pathological myocardial hypertrophy. However, they are far from conclusive and computational approaches combined with experimental studies are still necessary to further identify effective therapeutic strategies [7].

In this study, we explored myocardial hypertrophy based on omics databases and tools. Two datasets GSE18224 and GSE36074 from the GEO database obtained from mouse model were re-analyzed. After screening out the differential expressed genes (DEGs) in GSE18224, the gene ontology (GO) and kyoto encyclopedia of genes and genomes (KEGG) analysis were executed. Besides, the potential microRNAs (miRNAs) and transcription factors (TFs) targeting DEGs were predicted, and a protein-protein interaction (PPI) network was constructed for further analyzing the module. GSE36074 and our transverse aortic constriction (TAC) mouse model were successively applied to validate the expression of those genes in the module. Finally, the selected genes were evaluated for their correlation with the biomarkers of cardiac hypertrophy.

## Methods

### Bioinformatics datasets

Two microarray datasets GSE18224 [8] and GSE36074 [9] were obtained from the GEO database (https://www.ncbi.nlm.nih.gov/geo/), which is a public functional genomics data repository. In GSE18224, eight C57BL/6 wild-type mice underwent TAC to induce cardiac hypertrophy, and another eight were sham-operated. The expression profile of genes in the cardiac left ventricle was detected by GPL1261 platform. In GSE36074, seven wild-type C57BL/6 mice were subjected to aortic banding (AB) and five were subjected to sham operation to construct the non-failure cardiac hypertrophy model and normal model respectively. The expression profiling of genes in the left ventricular myocardium was also detected by GPL1261 platform.

**Fig. 1:**
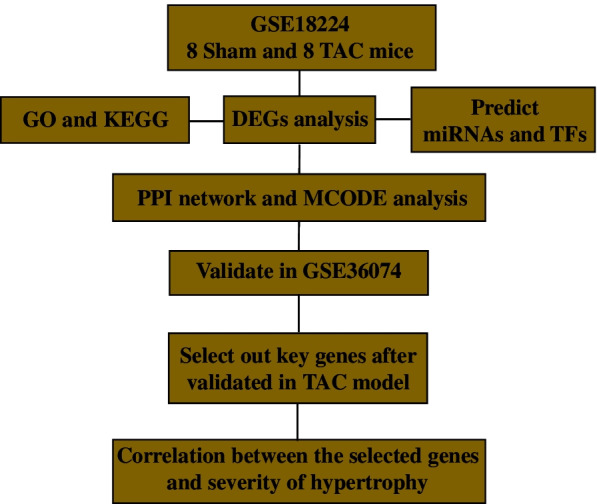
The research process of this study. TAC, transverse aortic constriction; DEGs, differentially expressed genes; GO, gene ontology; KEGG, Kyoto Encyclopedia of Genes and Genome; miRNAs, microRNAs; TFs, transcription factors; PPI, protein-protein interaction

The flow path of our study is displayed in Fig. 1.

### Identification of DEGs

GSE18224 and GSE36074 were both downloaded from the GEO database through the ‘geoquery’ package. The probes corresponding to multiple targets were removed. When a probe corresponding to the same molecule was encountered, only the probe with the largest signal value was retained. Subsequently, ‘limma’ package was used to analyze the difference in the expression of genes between the sham and TAC groups. The criteria for DEGs are as following: adjusted P value (adj.P.Val) < 0.05, and |log_2_ fold-change (FC)| ≥ 1.

### GO and KEGG enrichment analysis

KEGG database provides comprehensive knowledge for assisting biological interpretations of large-scale molecular datasets [10]. GO analysis is a bioinformatics resource that provides information regarding gene product function using ontologies to represent biological knowledge, and is mainly focused on three items: molecular functions (MF), biological processes (BP) and cellular components (CC) [11]. The GO and KEGG enrichment were analyzed with DAVID database (https://david.ncifcrf.gov/home.jsp), a web-accessible program that provides a comprehensive set of functional annotation tools for investigators to understand the biological meaning behind a large list of genes [12]. Finally, items of GO and KEGG with FDR < 0.05 were selected.

### Prediction of miRNAs and TFs targeting DEGs

TFs are a group of DNA-binding proteins with gene regulatory capabilities that regulate the expression of mRNAs at the transcriptional level [13]. MiRNAs are vital in regulating gene expression through binding with complementary target mRNAs and repressing their expression [14]. The easily accessible online web tool miRNET (https://www.mirnet.ca/) was used for predicting the miRNAs and TFs that potentially regulate the DEGs [15].

### Construction of protein-protein Interaction network

Protein-Protein Interaction (PPI) network of all the DEGs was constructed through the online web tool STRING (http://string-db.org). Cytoscape software was applied to re-visualize the whole PPI network [16]. Molecular Complex Detection (MCODE) is a method based on vertex-weighting by local neighborhood density and outward traversal from a locally dense seed protein to isolate the dense regions [17]. The MCODE plug-in of Cytoscape software was utilized to screen out significant modules among the PPI network. The parameters of MCODE were as following: degree cut-off = 2, node score cut-off = 0.2, k-score = 2, and max. depth = 100.

### Validation and selection of key genes

The module of the PPI network was regarded as vital in disease progression, and genes in the module were evaluated for their expression in dataset GSE36074. Those genes significantly differentially expressed in GSE36074 were selected and their expression level was displayed using the violin plot by GraphPad Prism 8.0.

In addition, based on dataset GSE36074, the expression levels of these genes and the recognized cardiac hypertrophy biomarkers including left ventricular weight/tibia length ratio (also called lvtindex), natriuretic peptide a (nppa), natriuretic peptide b (nppb), and myosin heavy chain 7 (myh7) [18, 19], were enrolled to calculate the correlation to each other by the online tool Hiplot (https://hiplot.com.cn) and GraphPad Prism 8.0 in Pearson method.

### Animal model

6-week-old male C57BL/6J mice were anesthetized by intraperitoneal injection of pentobarbital sodium (30 mg/kg). Subsequently, the chest of each mouse was opened at the second intercostals space, and the thymus glands were superiorly reflected. The transverse thoracic aorta between the brachiocephalic trunk and left common carotid artery were dissected and tied around the aorta against a 27-gauge needle, then ligated with a 6 − 0 nylon suture. The sham group mice were subjected to thoracotomy and aortic dissection without constricting the aorta. After surgery, the mice were housed at appropriate temperature and humidity, and maintained a 12 h dark-light cycle with free availability of food and water lasting for 2 weeks. Totally, there were 6 mice included in this study and equally divided into two groups.

 The animal study was approved by the Animal Ethics Committee of Medical School of Southeast University (NO: 20,211,201,005). All experimental methods were carried out in accordance with the animal ethics guidelines and regulations. This study was carried out in compliance with the ARRIVE guidelines.

### Echocardiography and histological staining

The mice were anesthetized and prepared for echocardiography by shaving the fur from neckline to mid-chest level. Cardiac anatomical and functional parameters were evaluated by 2-dimensional transthoracic echocardiography using the Vevo 2100 ultrasound system (Visualsonics). The indexes including end-diastolic left ventricular posterior wall thickness (LVPWd), ejection fraction (EF), and fractional shortening (FS) were collected.

Subsequently, the hearts were excised and the middle sections were taken out to be immediately fixed in 4% paraformaldehyde, embedded in paraffin and sectioned into 5 μm slices. The slices were stained with hematoxylin-eosin (H&E, Sigma) and fluorescein isothiocyanate-conjugated wheat germ agglutinin (WGA, Sigma) according to standard protocols to evaluate the cross-sectional area of cardiomyocytes.

### Quantitative real-time PCR for mRNAs

Total RNA was isolated from sample heart tissues with Trizol (Biosharp) according to the manufacturer’s instructions. cDNA was synthesized with HiScript III RT SuperMix for qPCR (+ gDNA wiper) (Vazyme). The ChamQ Universal SYBR qPCR Master Mix (Vazyme) was used in quantitative real-time PCR (qRT-PCR) with Quant Gene 9600 system (Bioer Technology). β-actin was used as internal control to calculate the relative mRNA expression in each sample by the 2^−∆∆Ct^ method. The primer sequences (Generay Biotech) of each gene were listed in Table 1.

**Table 1:** The primer sequences used for analysis

Genes	Primer sequences
Bgn	R: TGTGCTACTCACCTTGCTG
	F: TGGTGTCTGAACCTGAAGCC
Ctgf	R: TGCTGTGCATCCTCCTACCG
	F: CAGAGAGCGAGGAGCACCAA
Col5a2	R: ATGGGGTATGTAAAGCCTCAGC
	F: AGGACACAGGGGGCATTACT
Eln	R: CTGACTCGCGACCTAATCAA
	F: TCCTTGTCCTGTGGGTTTCC
Sox9	R: GGAAGGTAACGATTGCTGGG
	F: CCCTCCTCGCTGATACTGGT
Tgfb3	R: TGATGACCCACGTCCCCTAT
	F: CAGACTCCGAGGTCTCCTGA
Postn	R: GCAAACCACTTTCACCGACC
	F: CGTTGGTCCATGCTCAGAGT
Tgfb2	R: GCCAGGACACGAAAATCACG
	F: TCCCTCCCTCCTGTCACAAA
β-actin	R: GGGCAACCTTCCCAATAAAT
	F: CCATGTTCCAAAACCATTCC

### Western blot

Total protein from the ventricular tissue of the mouse models was extracted by RIPA buffer (KeyGEN BioTECH). The supernatant was obtained by centrifugation and the concentrations of protein were detected using a BCA Protein Assay Kit (KeyGEN BioTECH). Subsequently, the protein samples were separated by SDS-PAGE and transferred to PVDF membranes (Millipore). The blots were cut prior to hybridization with antibodies including anti-elastin (Affinity, 1:1000), anti-tgfb3 (Proteintech, 1:1000) and anti-β-Actin (ABclonal, 1:5000) overnight, and further cultured with HRP-conjugated secondary antibody (Biosharp). The immunoreactivity was visualized with an ECL kit (KeyGEN BioTECH). Finally, β-Actin was used as internal control to evaluate the relative expression of elastin and tgfb3. The signals were quantified by using ImageJ software.

### Statistical analysis

All data detected by qRT-PCR and western blot are expressed as the mean ± SD. Statistical difference between two groups was analyzed by unpaired Student’s t-test. SPSS 22.0 and GraphPad Prism 8 were used to calculate the *P* value, and *P* < 0.05 was considered statistically significant.

## Results

### Identification of DEGs in GSE18224

**Fig. 2:**
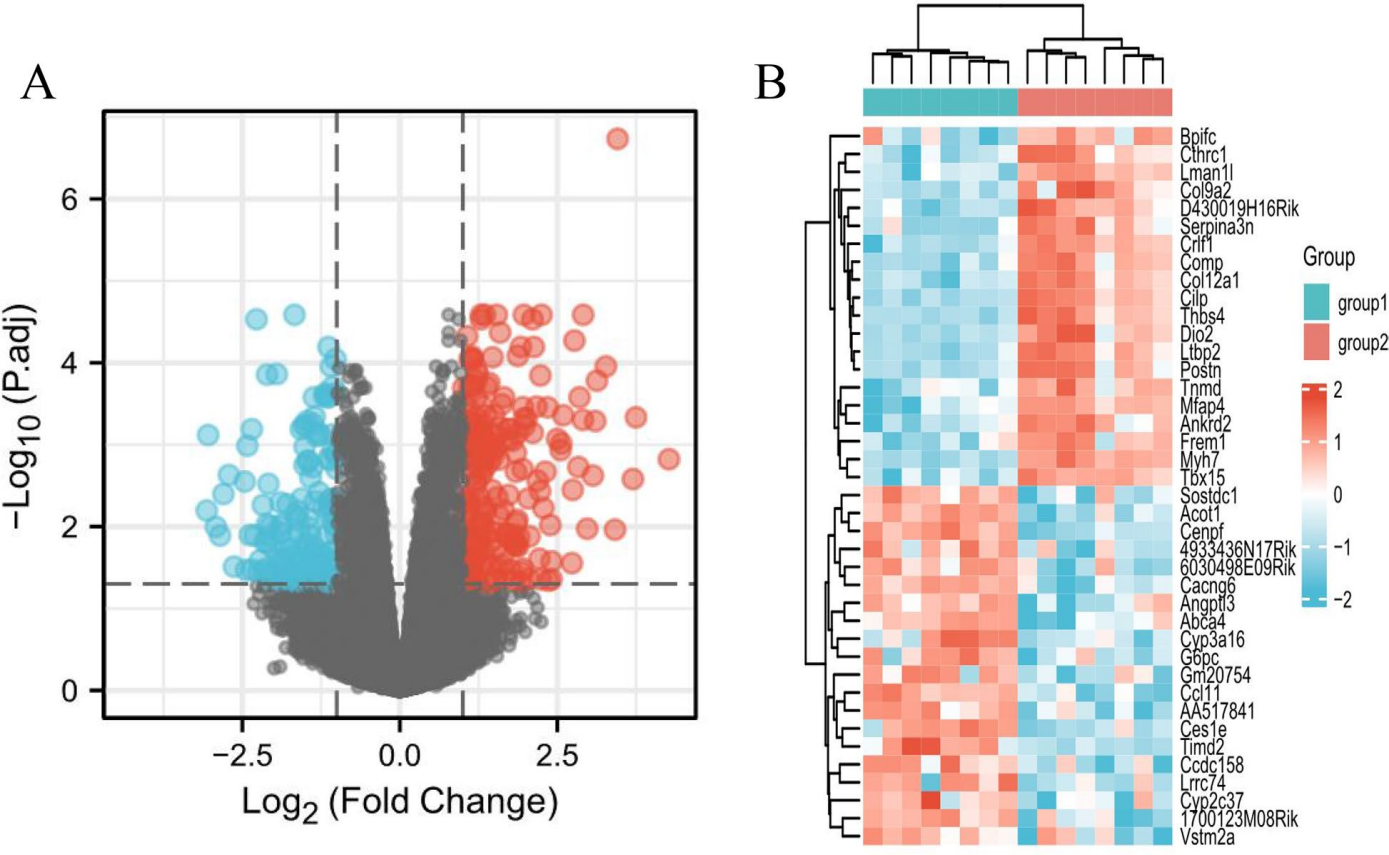
Identification of DEGs between controls and cardiac hypertrophy in GSE18224. **A** Volcano plot of DEGs in GSE18224. **B** Heatmap showed the relative level of the top 20 upregulated and downregulated DEGs in GSE18224

GSE18224 detected mRNA microarray of the heart’s left ventricle in eight mice with cardiac hypertrophy and eight corresponding control mice. After re-analyzing, there were 348 significantly dysregulated mRNAs in the hypertrophic myocardium, 203 of which were upregulated and 145 were downregulated (Fig. 2A). The top 20 upregulated and top 20 downregulated genes were selected to have their relative expression levels displayed in the form of a heatmap (Fig. 2B). The detailed information of the re-analysis results was summarized in Additional file 1.

### GO and KEGG enrichment analysis of DEGs

**Fig. 3:**
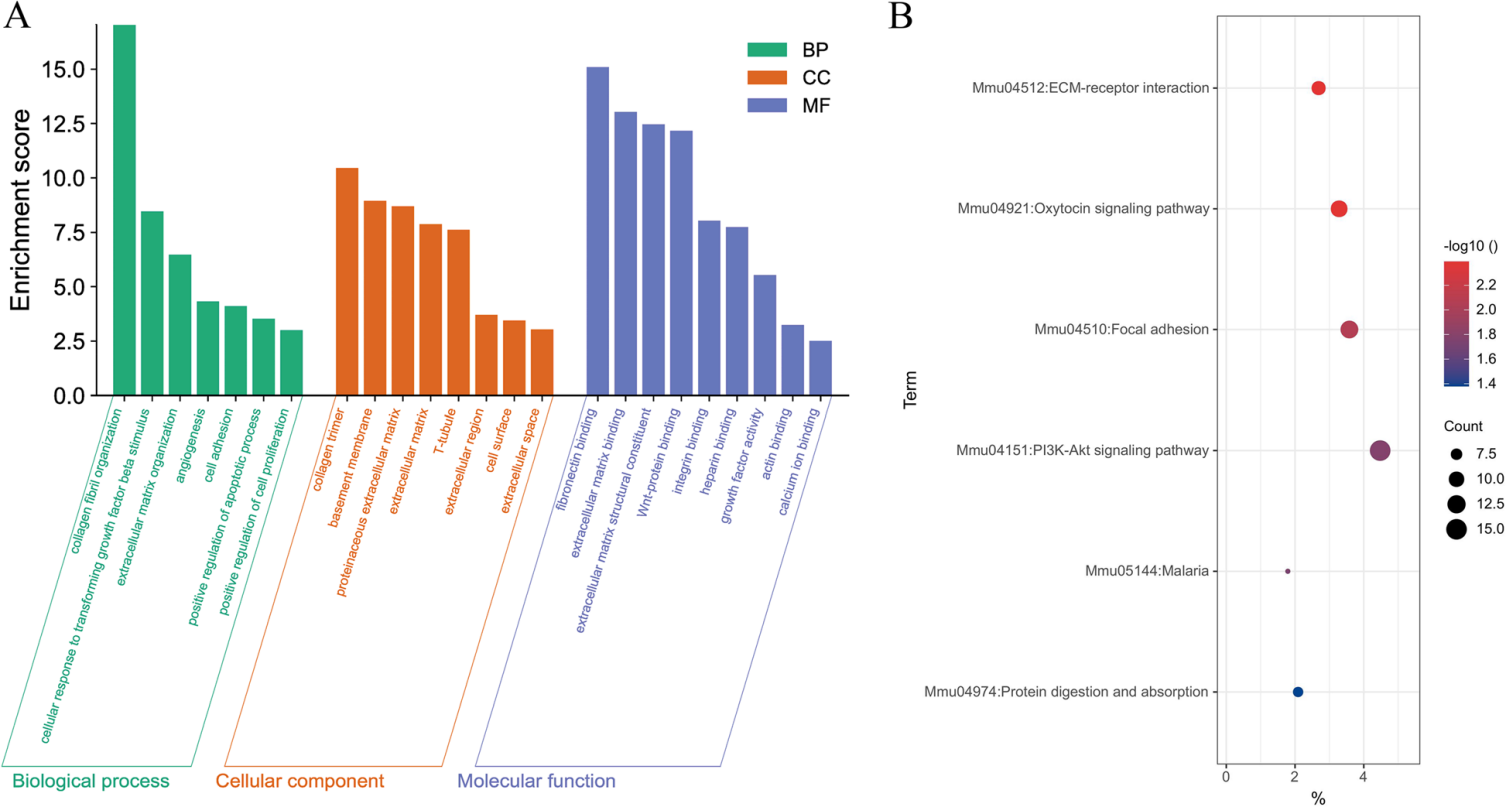
GO and KEGG analysis of DEGs. **A** GO research includes biological process, cellular component, and molecular function based on DEGs. The seven green items belong to biological process, the eight orange-red items belong to cellular components and nine purple items belong to molecular function. **B** KEGG enrichment results of the DEGs. The X-axis represents Gene-Ratio (%) and Y-axis represents items. The redder the color, the smaller the FDR value. The larger the size, the more genes it contains

The GO and KEGG analysis were performed based on the DEGs. As indicated in Fig. 3A, with regards to BP, the DEGs were mainly enriched in collagen fibril organization, cellular response to transforming growth factor-beta stimulus. In terms of CC, collagen trimer, basement membrane, and others were enriched, while fibronectin binding, and extracellular matrix binding, and extracellular matrix structural constituent were enriched by MF.

KEGG analysis showed that these DEGs were significantly enriched in several pathways, including ECM-receptor interaction, Oxytocin signaling pathway, and Focal adhesion (Fig. 3B).

### DEGs’ miRNAs and TFs prediction

**Fig. 4:**
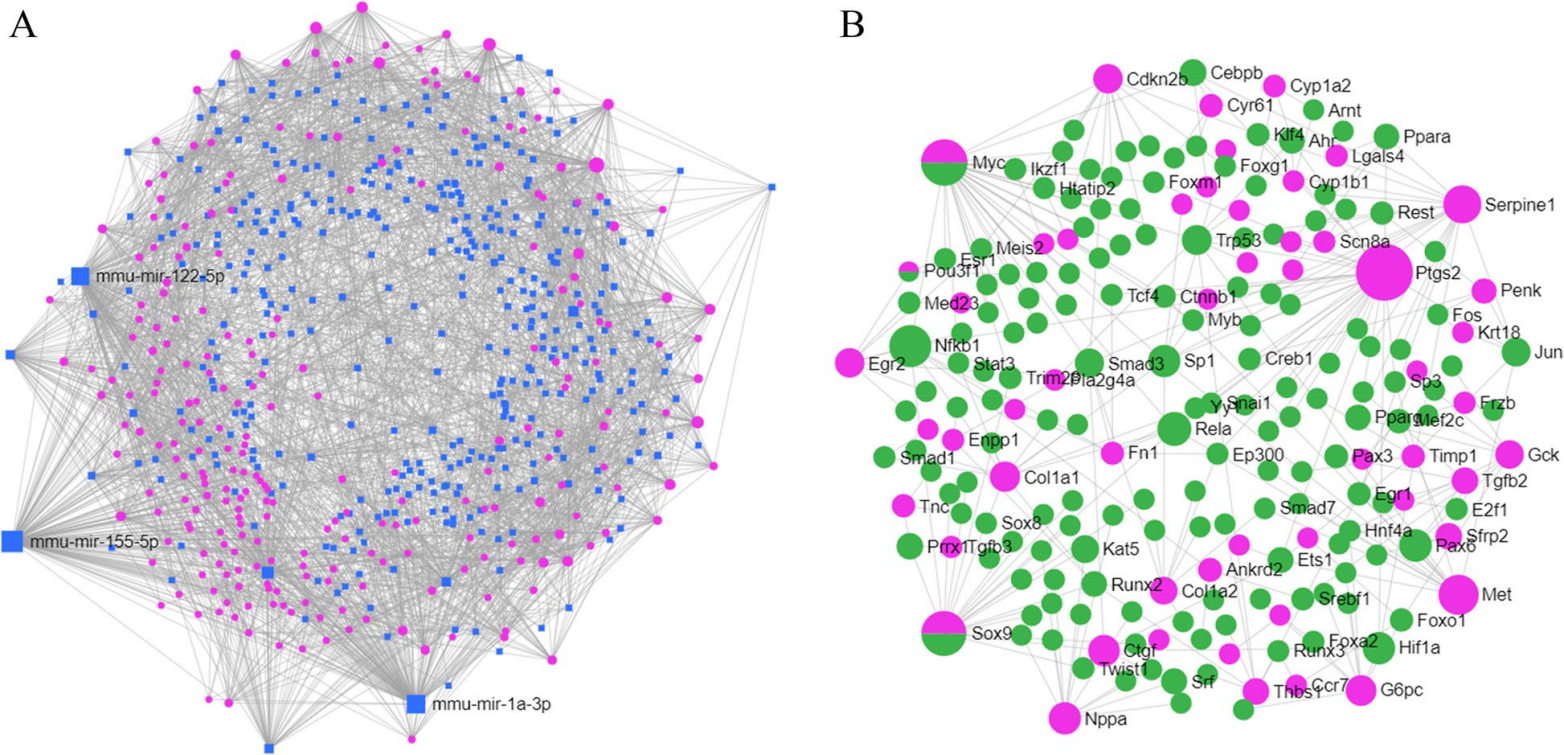
Prediction of the miRNAs and TFs. **A** The miRNAs-mRNAs network. Blue represents miRNAs and red represents mRNAs. **B** The TFs-mRNAs network. Green represents TFs and red represents mRNAs

The miRNAs and TFs of the DEGs were predicted using web tool miRNET. There were 387 miRNAs found to potentially regulate 257 of the 348 DEGs in the miRNA-mRNA network (Fig. 4A). Among these miRNAs, miR-122-5p, miR-155-5p, and miR-1a-3p had the most downstream target genes. The TF-mRNA network was also constructed, and 177 TFs were predicted to regulate 71 of the 348 DEGs (Fig. 4B). Besides, 3 DEGs, including Myc, Sox9, and Pou3f1 were also predicted to act as TFs in regulating other DEGs expressions. The detailed pairs of miRNA-mRNA and TF-mRNA were summarized in Additional file 2 and Additional file 3, respectively.

### Construction of PPI network and MCODE analysis

**Fig. 5:**
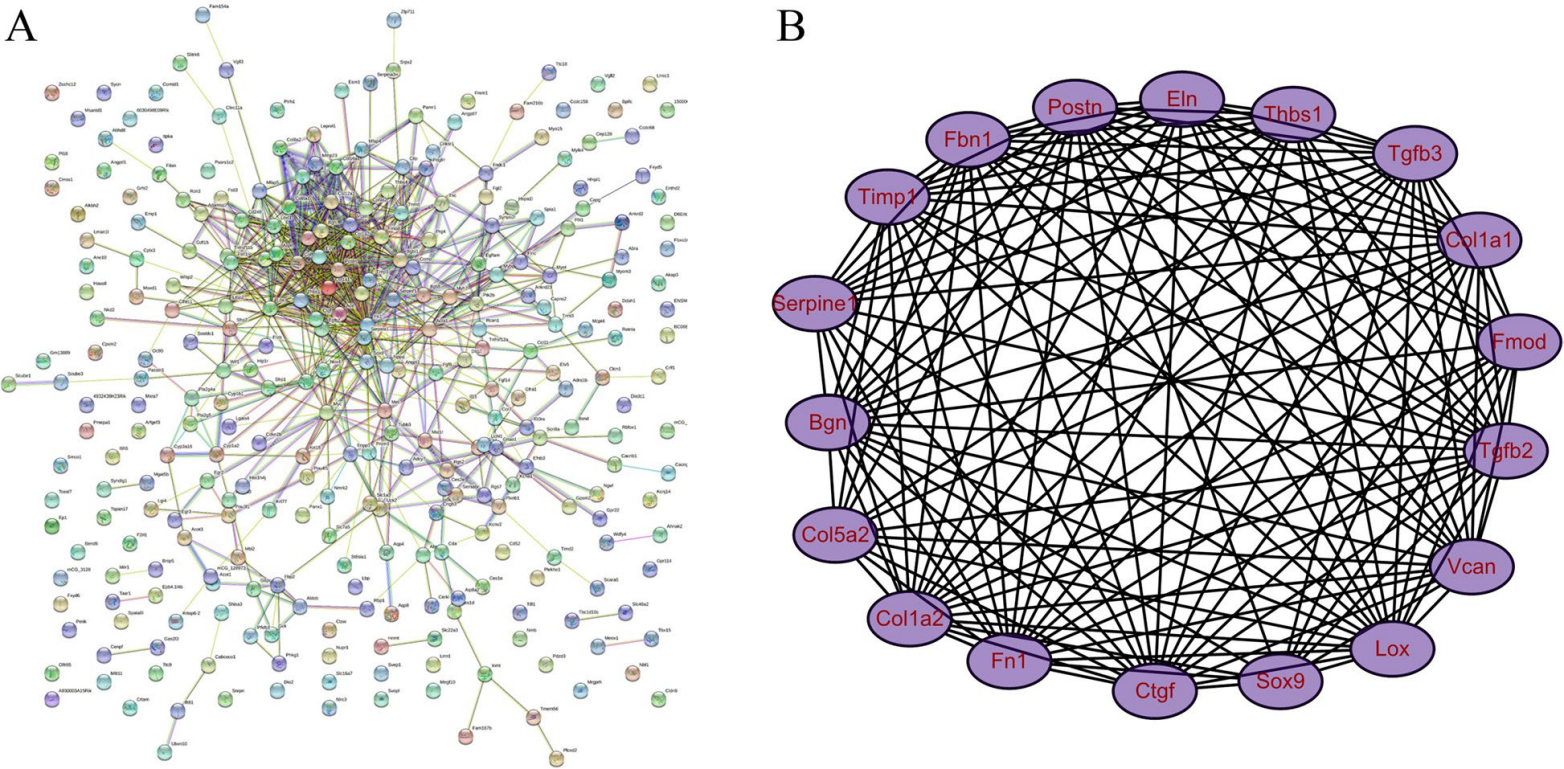
PPI network construction and modules analysis. **A** The PPI network. **B** MCODE analysis indicated the first module contained 18 genes and scored 16.353

The PPI network was constructed based on all the DEGs. Finally, the network was completed with 222 nodes and 770 edges (Fig. 5A). In order to filter out the module, we imported the network into the cytoscape software and analyzed it using the MCODE plug-in. The most significant module contained 18 nodes and 139 edges, and scored 16.353 points (Fig. 5B).

### Validation of the module with GSE36074 dataset

**Fig. 6:**
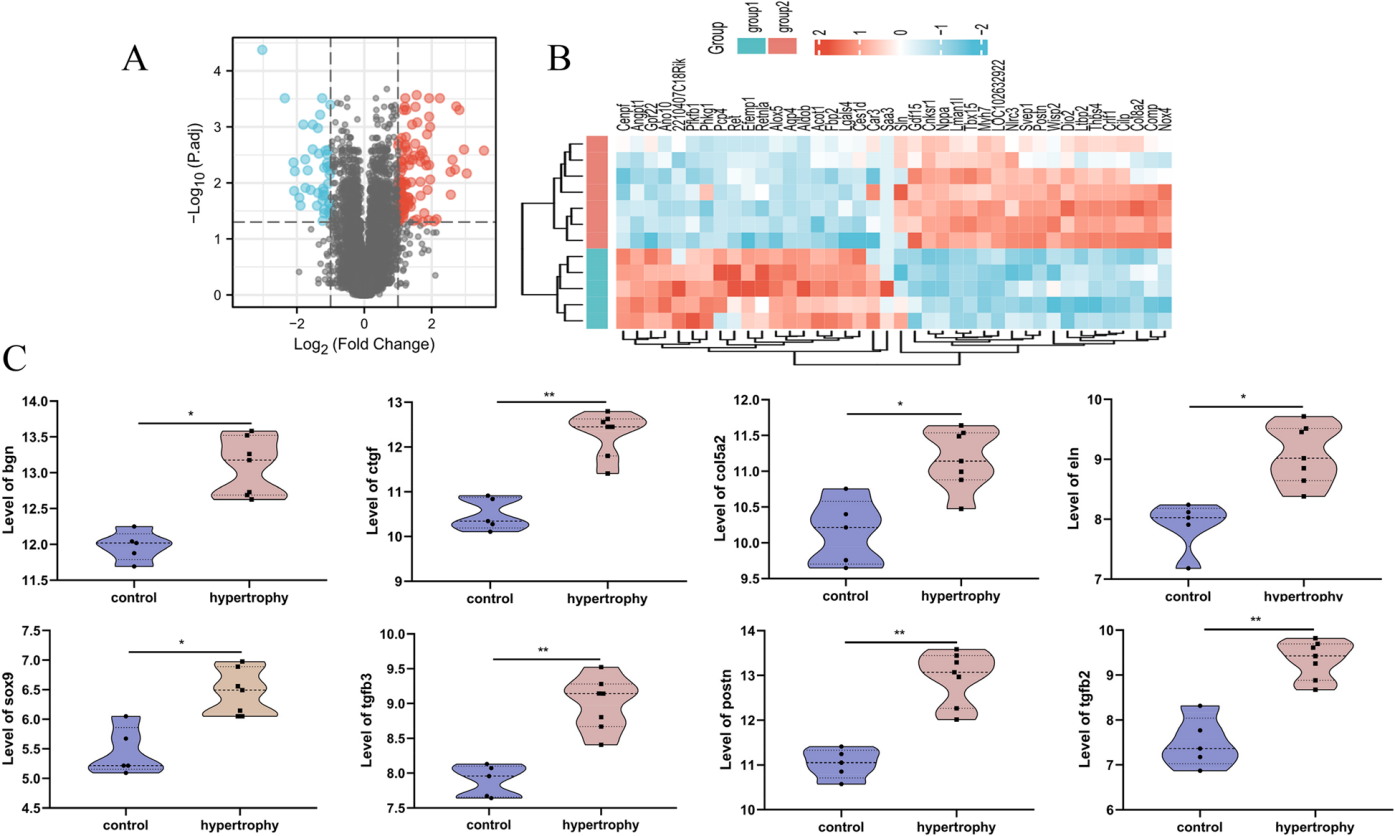
Validation of the dysregulated genes in MCODE-module by re-analysis GSE36074. **A** Volcano plot of DEGs in GSE36074. **B** Heatmap showed the relative level of the top 20 upregulated and downregulated DEGs in GSE36074. **C** Bgn, Ctgf, Col5a2, Eln, Sox9, Tgfb2, Tgfb3, and Postn were significantly upregulated in the myocardial hypertrophy group compared with the control group. * adj.P.Val < 0.05, ** adj.P.Val < 0.01

The GSE36074 dataset was re-analyzed to pick out the DEGs in the left ventricular sample of five control mice and seven cardiac hypertrophy mice without heart failure (Additional file 4). There were 104 upregulated genes and 44 downregulated genes (Fig. 6A). The relative level of the top 20 up/downregulated genes were displayed in the form of a heatmap (Fig. 6B). After verification, a total of 8 of the 18 genes, including Bgn, Ctgf, Col5a2, Eln, Sox9, Tgfb3, Tgfb2, and Postn in the module were found to be significantly differentially expressed in GSE36074, and were all upregulated in the hypertrophic myocardium (Fig. 6C). These 8 genes were regarded as vital in the progression of myocardial hypertrophy.

### Verification of the expression of the 8 genes in TAC mice model

**Fig. 7:**
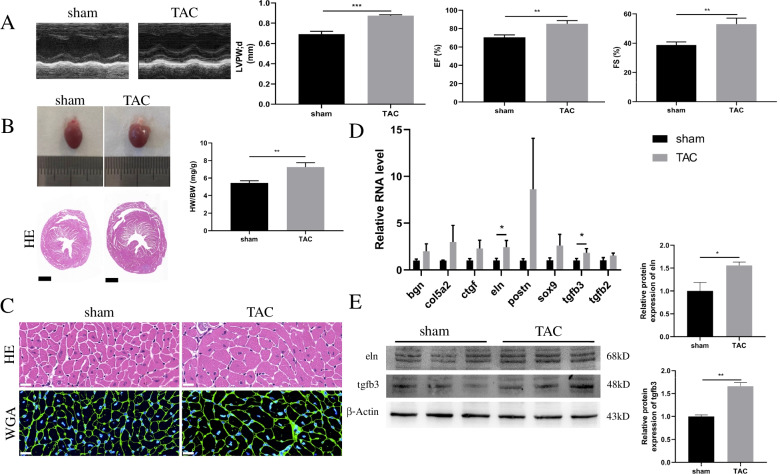
Validation of the expression of the genes in TAC mouse model by qRT-PCR. **A** Echocardiography results of sham and TAC mouse model. *n* = 3 in each group. **B** Heart morphology and the HW/BW ratio in sham and TAC groups. Scale bar = 1000 μm. *n* = 3 in each group. **C** HE and WGA staining of heart tissue in sham and TAC groups. Scale bar = 20 μm. *n* = 3 in each group. **D** qRT-PCR detected the expression of Bgn, Ctgf, Col5a2, Eln, Sox9, Tgfb2, Tgfb3, and Postn in hearts of sham and TAC groups. *n* = 3 in each group. **E** Western blot detected the expression of Eln and Tgfb3 in hearts of sham and TAC groups. The blots were cut prior to hybridization with antibodies during blotting. *n* = 3 in each group. * *P* < 0.05, ** *P* < 0.01, *** *P* < 0.001

To determine whether these 8 genes were truly dysregulated during the development of cardiac hypertrophy, we constructed the mice model of cardiac hypertrophy by TAC method. The ventricular wall thickness and cardiac function indexes including EF and FS increased significantly in TAC mice (Fig. 7A). Besides, the size of hearts, the HW/BW ratio (Fig. 7B) and the cross-sectional area of cardiomyocytes (Fig. 7C) increased in the TAC-induced hypertrophy mice model. All of these suggested that cardiac hypertrophy was induced successfully by TAC, same as in the GEO datasets. The results of qRT-PCR of the 8 detected genes indicated that Eln and Tgfb3 were significantly upregulated in hypertrophic myocardium compared with the normal myocardium (Fig. 7D). Besides, regardless of statistical significance, the other 6 genes all showed an increased expression trend, similar to their expression pattern in the datasets. This suggested that these genes, especially Eln and Tgfb3 are likely to play an important regulatory role in the disease process. To confirm the results revealed by PCR, we detected the protein level of Eln and Tgfb3 by western blot and found that both Eln and Tgfb3 proteins were also significantly increased in the hypertrophic heart compared to the respective controls (Fig. 7E, Additional file 5).

### Correlation between the 8 dysregulated genes and severity of hypertrophy

**Fig. 8:**
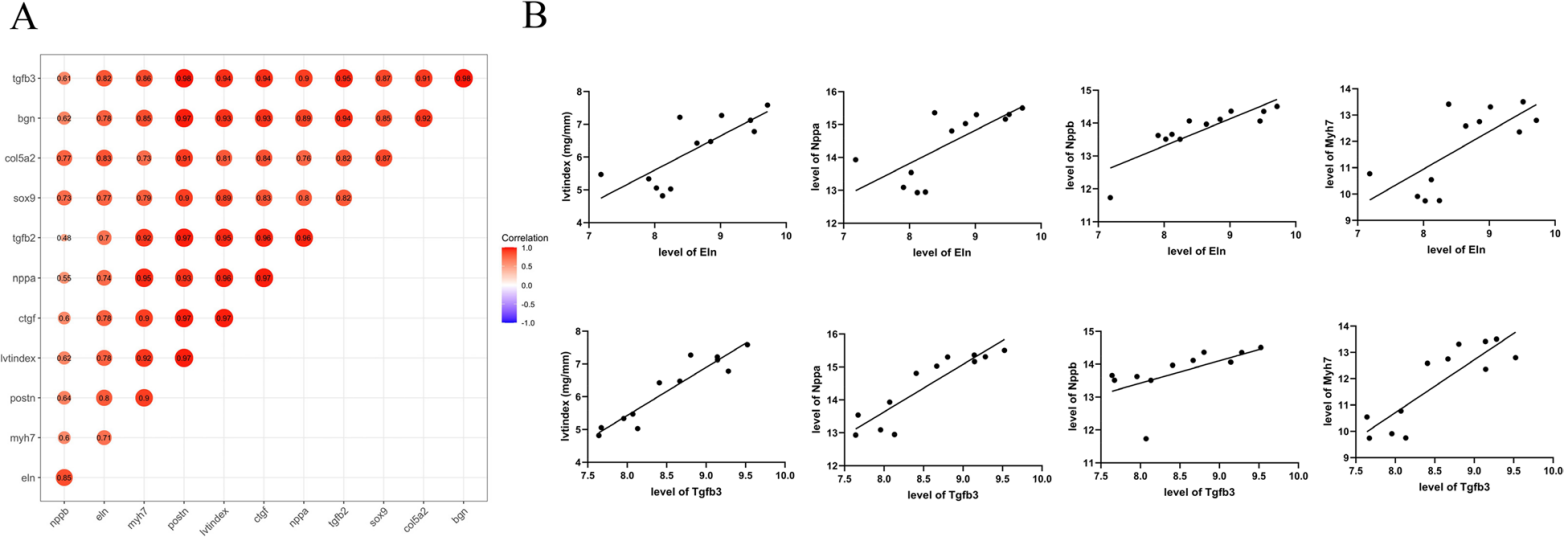
The correlation between 8 genes and biomarkers of myocardial hypertrophy in GSE36074. **A** The correlation-heatmap indicated the correlation coefficient among eight genes and four biomarkers, including lvtindex, nppa, nppb, and myh7. **B** The positive correlation between Eln and Tgfb3 and four biomarkers

To evaluate the correlation between the 8 differentially expressed genes with the severity of hypertrophy, the expression values of the genes and biomarkers, including lvtindex, Nppa, Nppb, and Myh7 were collected, and the correlation coefficient was calculated by Pearson statistic method. The results showed that all 8 genes were positively correlated with the four biomarkers (Fig. 8 A). More than that, the data distribution of Eln and Tgfb3 were displayed in the form of a scatter diagram, which indicated that these two key genes were significantly positively correlated with the severity of cardiac hypertrophy (Fig. 8B).

## Discussion

Hypertension is still the main reason for premature death worldwide [20]. As a major risk factor for cardiovascular disease, hypertension leads to various cardiac manifestations, including coronary heart disease and myocardial hypertrophy [21]. Although hypertrophic myocardium can maintain normal ejection function at an early stage, heart failure will inevitably occur as the disease progresses and gets out of control [2]. Therefore, timely intervention on left ventricular hypertrophy could potentially reduce the incidence of adverse cardiovascular events in patients with hypertension. Recently, several signaling pathways and a series of genes have been confirmed to be involved in the progression of myocardial hypertrophy [22]. However, it is still urgent to clarify the internal mechanism further and find the key pathogenic genes.

The progression of omics has promoted the revolutionary development of disease research [23]. In the present study, we obtained and re-analyzed the dataset GSE18224 that investigated the mouse model of myocardial hypertrophy from the GEO database. We finally screened out 203 upregulated and 145 downregulated genes. Among these dysregulated genes, several have been proved to have changes in expression and even participate in the development of this kind of disease. For example, MFAP4 was found to be increased in the cardiac remodeling of animal models induced by pressure overload and isoproterenol, which was similar to our results [24]. Besides, NOX4 was found to be upregulated in our analysis and confirmed the results by Matsushima S [25] that NOX4 expression in cardiomyocytes was increased in response to phenylephrine stimulation, and that cardiac-specific Nox4 knockout could relieve myocardial hypertrophy caused by pressure overload. These researches indicated that the DEGs screened out by bioinformatics should be considered reliable.

We subsequently performed GO and KEGG analyses to clarify the biological characteristic of the DEGs. These 348 DEGs were enriched in collagen fibril organization and cellular response to TGFB stimulus, and are mainly located in collagen trimer, basement membrane and extracellular matrix. Their function is strictly focused on fibronectin binding and extracellular matrix binding. The above results suggested that these DEGs were closely connected with fibrosis progression. Besides, the enriched signaling pathways include ECM-receptor interaction, which further confirmed that the fibrosis-related function participates in myocardial hypertrophy [26]. Cardiac fibroblasts are important participants in regulating the progression of cardiac hypertrophy [27]. Therefore, cardiac fibrosis should be regarded as the potential core of diagnosis and treatment.

In order to understand the possible mechanisms involved in DEGs expression changes, we predicted hundreds of miRNAs and TFs that regulate the DEGs. Of the predicted miRNAs, miR-122-5p, miR-155-5p, and miR-1a-3p targeted the most genes. In a recent study, miR-1a-3p was shown to play an antagonistic role in isoproterenol-induced heart failure [28], highly suggesting that the predicted miRNAs and the miRNA-mRNA pairs play a role in the disease process. Several recognized hypertrophic factors, including myc and sox9 [29, 30], which were upregulated in the dataset of the mouse myocardial hypertrophy model, were among the predicted TFs. In the complex TF-mRNA network, myc and sox9 were the transcription factors that target the largest number of downstream DEGs, indicating their crucial influence on the disease process. In fact, the other TFs could be potential key participants despite having non-statistically significant changes in their mRNA expression. The PPI network was constructed to focus on the DEGs’ internal interaction. A total of 222 genes were enrolled from all the 348 DEGs. The MCODE method was applied to analyze the network to pick out the subnetwork. Finally, the module with the highest score (16.353 points), consisting of 18 genes, including fmod, thbs1, col1a1, and sox9, was obtained from the whole network. These 18 genes were regarded as vital in the disease progression.

Subsequently, the reliability of the above 18 genes was assessed by re-analyzing the mouse model of myocardial hypertrophy dataset GSE36074. After performing the validation, we found that 8 genes, including Bgn, Ctgf, Col5a2, Eln, Sox9, Tgfb2, Tgfb3, and Postn, were significantly upregulated in hypertrophic myocardium. However, the other 10 genes were inconsistent with the results in the dataset GSE18224. We also constructed the TAC model and further detected the expression of the above 8 genes. Finally, our results revealed that the LVPW and cross-sectional area of cardiomyocytes of mice increased in response to pressure overload, indicating that the model of myocardial hypertrophy was successfully constructed. qRT-PCR analysis revealed that Eln and Tgfb3 were also significantly upregulated in the hypertrophic myocardium. Although there was no significant difference in expression of the other 6 genes, they all showed an upward trend in pathological cardiac hypertrophy, similar to their expression pattern in the two datasets. Western blot analysis of the cardiac tissue of the cardiac hypertrophy mouse model revealed significantly increased Eln and Tgfb3 protein expression levels. These results pointed out that the selected key genes may play critical roles in the development of cardiac hypertrophy.

Eln (elastin) is an extracellular matrix protein essential to the elasticity and resilience of multiple tissues, including artery vessels, lungs, etc. [31]. As a well-known regulator in multiple cardiovascular diseases, especially those affecting arterial blood vessels, Eln has been shown to lead to the formation of supravalvular aortic stenosis and arterial remodeling combined with hypertension due to its haploinsufficiency [32, 33]. However, the wild-type and Eln^+/*−*^ mice showed no significant difference in terms of left ventricular hypertrophy [34], suggesting that the lack of Eln in the myocardium may have a protective effect on hypertrophy under pressure overload and that the increased Eln expression could act as the pathogenic factor. Tgfb3, one of the three transforming growth factor-β (TGFβ) isoforms, is a vital driver of fibrotic disease pathogenesis [35]. These further confirmed the vital role of the fibroblast system in pathological myocardial remodeling revealed by GO and KEGG analysis. According to Chakrabarti, M. [36], the loss of Tgfb3 would cause a series of cardiovascular malformations, including abnormal ventricular myocardium or aortic/pulmonary trunk walls, outflow tract septal and alignment defects, and increased heart valve thickness, suggesting that Tgfb3 is essential for heart development. During disease progression, such as myocardial infarction, the expression of Tgfb3 will increase and act as a negative regulator of fibrosis [37]. However, in a rat model of aortic constriction (AC) induced cardiac hypertrophy, immunoblotting revealed that Tgfb3 levels decreased continuously in left ventricular tissue of hypertrophy rats starting from the 3rd-day post-operation [38], which seems to contradict our research. This may be caused by the difference in model species and evaluation methods. Therefore, a more in-depth analysis is needed in the future to clarify the role of Tgfb3 in the process of myocardial hypertrophy.

The expression of Eln and Tgfb3 was used to calculate their correlation with the severity of cardiac hypertrophy. We found that the expression of Eln and Tgfb3 were both positively correlated with the level of hypertrophic biomarkers, suggesting that the higher the level of Eln or Tgfb3, the more severe the myocardial remodeling. Considering that these two factors are exocrine proteins secreted by tissue/cells [39, 40], they are highly likely to be valuable circulating biomarkers to judge the progression of pathological myocardial hypertrophy.

Among all the 8 genes, Col5a2 has never been clarified to have a direct or indirect relationship with cardiac hypertrophy. Col5a2, a subtype of collagen V, is responsible for regulating collagen production in fibrotic tissue [41]. During the past decades, the biological role of col5a2 in a variety of diseases has been revealed. For example, in multiple cancers such as bladder cancer, gastric cancer and colorectal cancer, increased expression of Col5a2 was correlated with poor clinical outcomes and survival of patients [42–44]. Besides, Col5a2 could promote proliferation and invasion of prostate cancer and predict recurrence-free survival [45]. There is also a close connection between Col5a2 and non-cancerous diseases. Yang F, et.al [46] found that Col5a2 is decreased in steroid-induced necrosis of the femoral head. In cardiovascular diseases, Col5a2 has an equally indispensable role. For example, during arteriogenesis, Col5a2 was found to be downregulated in growing collaterals and targeted by miR-143-3p, thus contributing to outward vessel remodeling [47]. In mouse model of Col5a2 haploinsufficiency, the severity of abdominal aortic aneurysms was significantly raised, inducing aortic arch ruptures and dissections [48]. A study involving diseased hearts showed that Col5a2 expression was increased in myocardial infarction samples compared to the control samples and played an excellent role in distinguishing myocardial infarction [49]. Our analysis indicated that Col5a2 is likely to be upregulated during the development of myocardial hypertrophy. However, whether and how it performs a function in the process of this disease is to be solved.

## Conclusions

In summary, we discovered a series of abnormally expressed genes in the development of cardiac hypertrophy and screened out the key genes, including Eln and Tgfb3. The level of Eln and Tgfb3 were significantly increased during the progression of cardiac remodeling and have a positive correlation with the severity of myocardial hypertrophy. It is worth exploring the clinical role of these two key genes in cardiac hypertrophy.

## Supplementary Information

**Additional file 1.**

**Additional file 2.**

**Additional file 3.**

**Additional file 4.**

**Additional file 5.**

## Data Availability

The data analyzed during the current study are available in the GEO database (https://www.ncbi.nlm.nih.gov/geo/) with accession number GSE18224 and GSE36074.
