# Supplementary material for: Upregulation of key genes Eln and Tgfb3 were associated with the severity of cardiac hypertrophy
Source: BMC Genomics. 2022 Aug 14;23:592. doi: 10.1186/s12864-022-08778-0 (PMC9375926; doi:10.1186/s12864-022-08778-0)
Supplement: Supplementary file 5 — Additional file 5. [file 12864_2022_8778_MOESM5_ESM.pdf]

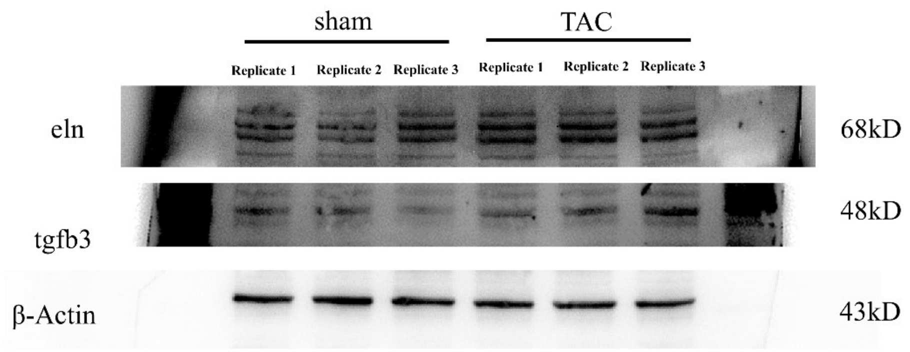

Additional file 5. The target bands and western blots of eln and tgfb3. β-Actin was used as internal control. The blots were cut prior to hybridization with the antibodies used for these two proteins. n = 3 in each group.
